# “Cow’s Hoof” (*Bauhinia* L., Leguminosae): A Review on Pharmacological Properties of Austral South American Species

**DOI:** 10.3390/plants12010031

**Published:** 2022-12-21

**Authors:** Renée Hersilia Fortunato, María Jimena Nores

**Affiliations:** 1 Instituto de Botánica Darwinion (CONICET/ANCEFN), Labardén 200, Acassuso 1641, Argentina; 2 Facultad de Ciencias Exactas, Físicas y Naturales, Instituto Multidisciplinario de Biología Vegetal (CONICET—Universidad Nacional de Córdoba), UNC, Vélez Sarsfield 1611, Argentina

**Keywords:** analgesic, anti-inflammatory, *Bauhinia*, diabetes, hypoglycemic, medicinal

## Abstract

The genus *Bauhinia s.l.* (Leguminosae), known as cow’s hoof, unha de boi or pata de vaca, has been used in traditional medicine worldwide. The aim of the present review is to summarize the studies published on the biological activity of the main native medicinal species reported in austral South America. Of the 14 species present in the region, 10 are consumed as leaf infusions to regulate glucose and lipid metabolism, as well as used for their anti-inflammatory and analgesic effects and to treat various diseases. Pharmacological properties have been recorded in seven species. Antioxidant, anticoagulant, antihypertensive, diuretic, antimicrobial and antitumor properties have been reported in *B. forficata*. Together with *B. holophylla*, they are important for their antidiabetic properties, since several studies indicate their effectiveness as a hypoglycemic agent. *B. bauhinioides* is distinguished for its anti-inflammatory and antithrombotic activities and *S. microstachya* for its analgesic properties. Anti-ulcer and wound healing activities recorded in *B. holophylla* and *B. ungulata*, respectively, are of particular interest. Most of the species possess antitumor activity. The antioxidant capacity of flavonoids and other bioactive compounds make these plants good candidates to assist or treat various alterations related with oxidative stress, such as diabetic complications. Thus, these species constitute promising targets for new bioactive substance research and phytotherapy.

## 1. Introduction

The species of the genus *Bauhinia s.l.* (Leguminosae, Cercidoideae), popularly known as cow’s hoof, cow’s paw, orchid trees, pata de vacca, unha de boi, falsa caoba, pezuña de vaca or pata de vaca, have been traditionally employed by different communities all over the world for medicinal purposes. Bilobed or bifoliolate leaves are consumed in infusions to treat diabetes mellitus, pains, inflammation and several diseases. The genus has promising medicinal potential, since experimental studies have provided evidence of its therapeutic properties [1,2,3].

In austral South America, 14 native species of trees, lianas and shrubs inhabit forests of Argentina, Paraguay, Uruguay and the southern states of Brazil [4,5,6]. In the region, 10 of these species are popularly used mainly to regulate glucose and lipid metabolism, but also as anti-inflammatory and analgesic agents and for treating digestive, kidney and urinary disorders, among others (Table 1; see references therein). Native American, rural and urban populations consume some of these species as crude herbs or industrialized herbal medicines composed mainly of entire or broken dried leaves and often young stems, pods and flowers [7,8,9,10,11,12]. In general, plant materials are harvested from their natural habitats and prepared in aqueous infusions or teas, decoctions and tinctures [1,9,13]. In addition, *B. forficata* leaves are added to mate or chimarrão—a drink prepared with leaves of *Ilex paraguariensis* A. St.-Hil.—or used as an alcoholature [13,14]. *Schnella microstachya* is also consumed after meals in a preparation of leaves with cachaça, a local sugar cane brandy, termed “garrafada” [15]. In the case of commercial samples that are sold in open markets or herbalist shops, they sometimes present strange materials that are often labeled and traded by using common names, generic names or incorrect names and the identification of species from vegetative or fragmented material becomes complicated; thus, the botanical quality of the samples is not always adequate [16,17,18]. While *B. forficata* has been more extensively studied [19,20,21,22,23], the pharmacological properties of most of the regional species are less well known.

In this work, we have compiled records of bioactive properties of austral South American species reported in the literature in order to contribute to the knowledge of these promising medicinal entities.

## 2. Methods

**Figure 1:**
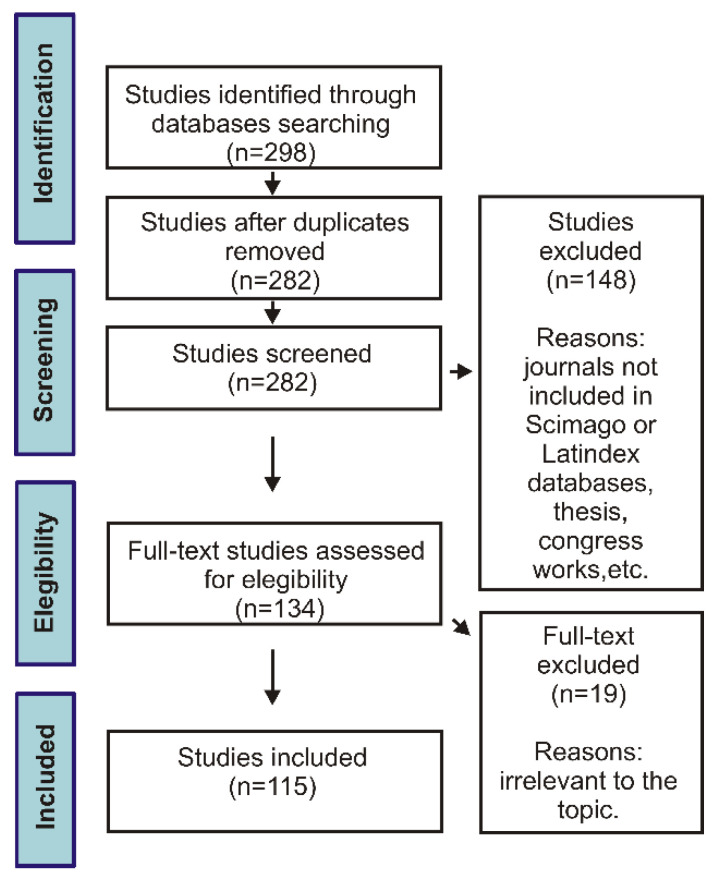
Literature screening procedure (flow chart).

A review of the literature available on the bioactivity of austral South American *Bauhinia* was conducted in January (2021) in the scientific databases Google Scholar and PubMed (Figure 1). The inclusion criteria were: (i) peer-reviewed articles published in journals listed in ScimagoJR [47] and/or indexed in Latindex [48]; (ii) English, Spanish and Portuguese literature; (iii) coverage time from 2000 to 2020. The exclusion criteria were: (i) studies published in non-indexed journals, theses, dissertations, conference proceedings and congress abstracts; (ii) patents; (iii) studies focusing on the structure of compounds or optimization of analytic methodologies. 

The search terms used in combination were “*Bauhinia*”, the scientific binomial of each species, “biochemical properties”, “chemical composition”, “diabetes”, “medicinal”, “pharmacological”, “phytochemical”. We analyzed the following 19 taxa: *B. affinis* Vogel; *B. amambayensis* Fortunato; *B. argentinensis* Burkart var. *argentinensis*; *B. argentinensis* Burkart var. *megasiphon* (Burkart) Fortunato; *B. bauhinioides* (Mart.) J.F. Macbr.; *B. campestris* Malme; *B. cheilantha* (Bong.) Steud.; *B. forficata* Link. subsp. *forficata*; *B. forficata* Link. subsp. *pruinosa* (Vogel) Fortunato & Wunderlin (under the name *B. candicans* Benth.); *B. hagenbeckii* Harms; *B. holophylla* (Bong.) Steud.; *Schnella microstachya* Raddi var. *microstachya* (under the name *B. microstachya* (Raddi) J.F. Macbr. var. *microstachya*); *S. microstachya* var. *massambabensis* (Vaz) Trethowan & R. Clark (under the name *B. microstachya* (Raddi) J.F. Macbr. var. *massambabensis* Vaz); *B. mollis* (Bong.) D. Dietr. var. *mollis*; *B. mollis* (Bong.) D. Dietr. var. *notophila* (Griseb.) Fortunato; *B. rufa* (Bong.) Steud.; *B. ungulata* L. var. *cuiabensis* (Bong.) Vaz; *B. ungulata* L. var. *ungulata*; *B. uruguayensis* Benth. Subspecies and varieties were discriminated. In the case of *B. forficata*, taxa were cited following the nomenclature used by the authors in their papers.

During the search, 298 references where evaluated. After eliminating duplicates, the literature was selected on the basis of inclusion/exclusion criteria. In the review, 117 references were included.

## 3. Biological Activity

**Figure 2:**
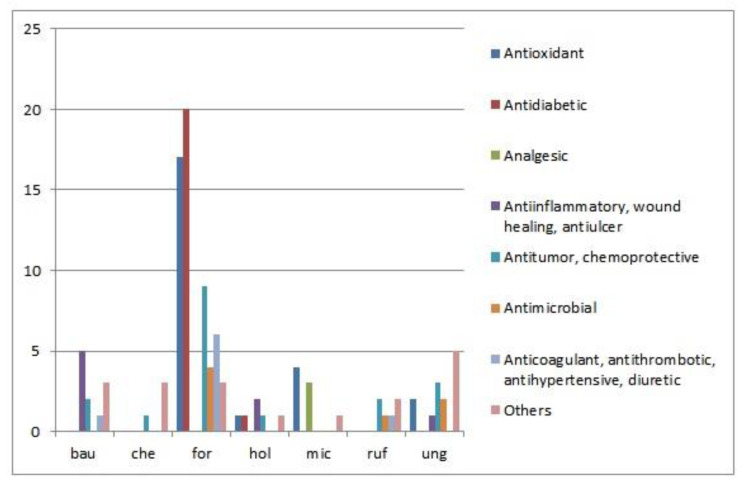
Biological activity of austral South American *Bauhinia*. Graphical representation of the number of published studies that reported each biological activity. The activities are grouped into eight categories discussed in Section 3.1, Section 3.2, Section 3.3, Section 3.4, Section 3.5, Section 3.6, Section 3.7 and Section 3.8 in the text. bau*—bauhinioides*; che—*cheilantha*; for—*forficata*; hol—*holophylla*; mic—*microstachya*; ruf—*rufa*; ung*—ungulata*.

Biological activity has been reported for seven species: *Bauhinia forficata*, *B. ungulata*, *B. bauhinioides*, *S. microstachya*, *B. holophylla*, *B. rufa* and *B. cheilantha*. These activities are grouped into eight categories discussed in Section 3.1, Section 3.2, Section 3.3, Section 3.4, Section 3.5, Section 3.6, Section 3.7 and Section 3.8 in the text (Figure 2; Table 2); in particular, antidiabetic properties and related activities are shown in Table 3. Antidiabetic, antioxidant and antitumor and chemoprotective activities are the main categories published in the analyzed literature. The most studied taxon is *B. forficata*, which is one of the 71 plants belonging to the National Relation of Medicinal Plants of Interest of the Single Health System in Brazil [24]. Most of the activities are attributed to flavonoids, such as kaempferol, quercetin or myricetin derivatives, which have been characterized in five species with differential metabolite profiles (Table 4). Chemical constituents include terpenoids, alkaloids, steroids, phenolic acids and fatty acids, among others (Table 4).

### 3.1. Antioxidant Activity

Antioxidant activity can be relevant in diseases that involve an increased production of free radicals or impaired antioxidant defenses, such as in diabetes mellitus and its complications, cardiovascular diseases, cancer, inflammation and aging [154,155]. Antioxidant activity of different extracts has been demonstrated in vitro and in vivo in *B. forficata*, *B. ungulata*, *S. microstachya* and *B. holophylla* (Table 2). Among the most relevant results, *B. forficata* subsp. *pruinosa* leaf tea (1 mg/mL for 21 days) exerted a hepatoprotective effect, modulating the increase in liver oxidative damage and reducing NADPH quinone oxidoreductase 1 expression levels in the pancreas in streptozotocin-induced diabetic mice [84]. In pregnant streptozotocin-diabetic rats, treatment with *B. forficata* aqueous extracts (500–1000 mg/kg for 20 days) maintained a reduced glutathione concentration in the blood and contributed to a decreased incidence of fetal visceral anomalies in treated diabetic rats compared with the untreated ones [76]. Sampaio et al. [88] proposed a protective effect of flavonoids on the male genital system and they reported a reduction in malondialdehyde levels—a biomarker of lipid peroxidation—in testicular and epididymal tissues obtained from rats treated with alcoholic extracts (0.1 mL/10 g for 30 days) compared with controls. Peroza et al. [80] detected antioxidant activity in a model of orofacial dyskinesia in rats induced by antipsychotics (see Section 3.8). Interestingly, Pedrete et al. [156] detected oxidative stress-related proteins involved in peroxide degradation, such as succinate semialdehyde dehydrogenase 2-cis peroxiredoxin and alcohol dehydrogenase, in *B. forficata* proteome. The high-antioxidant capacity found by Mansur et al. [105] in various leaf extracts and fractions of *B. microstachya* var. *massambabensis* led the authors to formulate an oil-in-water photoprotective emulsion for cosmetic use, containing sunscreens/1% leaf extract [106]. Assays with different formulations, tested in vitro and in vivo with human volunteers, demonstrated that leaf extracts contribute to enhance the sun protection factor. Finally, a *B. holophylla* leaf hydroalcoholic extract (150 mg/kg) significantly increased the level of glutathione and the activities of glutathione peroxidase and glutathione reductase in rat stomachs with ethanol-induced gastric ulcers [97]. These antioxidant effects have been attributed to phenolic components, mostly flavonoids, such as kaempferitrin, quercetin and rutin (Table 4; e.g., [84,89,106,114]).

### 3.2. Antidiabetic Properties and Related Activities

Most taxa in the region are used in traditional medicine to prevent or treat diabetes mellitus (Table 1), a group of metabolic diseases characterized by hyperglycemia resulting from defects in insulin secretion, insulin action or both [157]. *Bauhinia forficata* is the most studied taxon, while *B. holophylla* has been recently explored (Figure 2; Table 3). Research has been conducted on different leaf extracts in vitro and orally administered in normoglycemic and hyperglycemic animal models. For the other taxa, no studies have been identified that clearly document their effective medicinal properties.

#### 3.2.1. Bauhinia Forficata

Experimental diabetes induced by alloxan (ALX) or streptozotocin (STZ) produced hyperglycemia that was significantly reduced in acute, subacute and chronic treatments with leaf extracts in diabetic rats [68,125,132,134]) and rabbits [127]; moreover, acute treatments exerted hypoglycemic activity in normoglycemic rats and mice [125,131]. Furthermore, the extracts exerted a hypoglycemic effect in other models of hyperglycemia, such as the one induced by scorpion venom in rats [130]. On the contrary, it should be noticed that various chronic treatments failed to control glycemia [76,84,133]. Some treatments improved physiological or metabolic variables typically altered in the diabetic state, leading to a reduction in urine volume [127] and the urinary urea [124], as well as proteinuria and urine pH [134]. Regarding lipid metabolism, Lino et al. [129] demonstrated lipid-lowering properties with reduction in triglycerides (78–91%), total cholesterol (28–50%) and high-density lipoprotein (HDL) (27–68%) compared with diabetic controls, but other studies did not find changes in serum levels of different lipids [123,124,126,133]. Weight recovery was observed in treated diabetic animals by Curcio et al. [134] and this parameter, along with increased food and liquid intake, were not modified by other treatments [123,124,133]. Plant extracts reduced protein glycation in vitro; an activity that is important to decrease formation of advanced glycation end products (AGEs) produced during type 2 diabetes [86,89]. 

Regarding the hypoglycemic mechanism, an insulin-like effect has been hypothesized through peripheral glucose consumption, the regulation of key metabolizing enzymes, a delay in insulin catabolism or an increase in residual insulin efficiency or the inhibition of glucose reabsorption by the kidney (e.g., [74,78,125,135]). Increased glucose transport on peripheral tissues has been proved in isolated gastric glands [135]. Extracts inhibited in vitro enzymes such as α-glucosidase—which catalyzes the final step in the digestion of carbohydrates—and α-amylase and lipase, associated with postprandial hyperglycemia and hyperlipidemia in this metabolic disorder [78,86,89]. The increment of glycogen levels [126] suggests a regulation of glycogenolysis. Flavonoids, and particularly kaempferitrin (kaempferol 3,7-dirhamnoside), a major compound in both subspecies *B. forficata* leaves, are the main candidates for hypoglycemic action (Table 3 and Table 4). Acute treatment with purified kaempferitrin produced a significant hypoglycemic effect in diabetic [74,128] and normal rats [74]. Kaempferitrin favored peripheral glucose consumption, stimulating the glucose uptake in normal rat soleus muscle in vitro [128], involving synthesis, translocation and activation of the glucose transporter GLUT4 [136]. Glucose transport is mediated by the insulin signaling pathway that involves PI3K (phosphoinositide 3-kinase)-PKB (protein kinase B) and atypical PKC (protein kinase C) activation, together with the p38 MAPK (mitogen-activated protein kinase) pathway, which stimulates the expression of transporters or proteins from the insulin phosphorylation cascades [136]. Moreover, kaempferitrin stimulated in vitro glycogen synthesis and increased glycogen content in skeletal muscle [136]. On the other hand, Prasad et al. [158] showed that this compound from *B. acuminata* inhibits GLUT4 translocation. The role of kaempferitrin in glucose metabolism is demonstrated in other plants, where it stimulates 6-phosphofructo-1-kinase—the enzyme essential for controlling glycolysis—in the liver of diabetic mice, and other enzymes such as hexokinase and pyruvate kinase in myoblast cells [159]. Other compounds with potential hypoglycemic properties are the flavonoid rutin present in all subspecies of *B. forficata* and the alkaloid trigonelline identified in *B. forficata* subsp. *pruinosa* ([138]; Table 4). Pedrete et al. [156] identified enzymes of the glucose metabolism such as glyceraldehyde-3-phosphate dehydrogenases involved in glycolysis and gluconeogenesis and in controlling glucose levels and did not detect insulin-like proteins in *B. forficata* proteome. 

Three clinical studies have been conducted in pre-diabetic or/and type 2 diabetic volunteers with *B. forficata* infusions (3–10 months), with effects neither in lowering fasting plasma glucose levels [137,138] nor in postprandial glycemia [139]. Auspiciously, a statistically significant reduction in the percentage of glycated hemoglobin (0.57% and 0.25%) was detected after the treatment in diabetic patients [138,139], respectively; these studies did not include control groups in their designs. Conversely, no reduction in glycated hemoglobin values was reported by Pozzobon et al. [137]. Mariángel et al. [139] detected a significant reduction of triglycerides (26%) and total cholesterol (9%), been not clinically significant; the changes in the lipid profile are attributed to trigonelline and rutin or other quercetin derivatives and flavonoids. These studies are based on small samples and the evidence is not conclusive. Thus, further researching is necessary to ensure clinical effects of infusions in the prevention or complementary treatment of diabetes in patients. 

#### 3.2.2. Bauhinia holophylla

Camaforte et al. [141] demonstrated hypoglycemic and hypolipidemic activities when administering ethanolic extracts to STZ diabetic mice. Fasting blood glucose decreased significantly (up to 50%), glucose tolerance improved and hepatic glycogen levels increased. The extracts also modulated gene and protein expressions of enzymes involved in carbohydrate metabolism. Then, the authors proposed that the extracts stimulate glycogenesis in the liver by inhibition of GSK3-β (glycogen synthase kinase 3β) through the PI3K/Akt (protein kinase B) pathway and inhibit gluconeogenesis. Furthermore, they favor the glucose uptake in the muscle by activation of the PI3K/Akt pathway. In addition, they favor the increase of the glucose transporter 4 (GLUT4) expression, stimulate glycogenesis in this tissue and inhibit intestinal α-glucosidase enzymes. In contrast, Pinheiro et al. [140] have previously reported non hypoglycemic effects in non-diabetic and STZ female diabetic rats and a possible toxic effect of this plant. HDL-cholesterol levels decreased in the treated diabetic group (40.2 ± 5.7 mg/dL) compared with untreated ones (61.9 ± 10.2 mg/dL). The authors warn about liver damage, since a reduction in the body weight of the treated diabetic rats was detected compared with the non-treated ones, along with increased activities of the hepatic enzymes alanine aminotransferase and aspartate aminotransferase. It is interesting to mention that, in this species, kaempferitrin has not been found; instead, flavonoid derivatives of quercetin, myricetin, luteolin and kaempferol and isorhamnetin were reported (Table 4). 

Discrepancies observed in the results in both species may be due to different variables. The plant-extraction method and the solvent used influence the chemical composition of the resultant extract and subsequently its biological activity. For instance, the non-extraction or absence of kaempferitrin in the extracts could explain negative or weak results found by Ferreres et al. [78], Farag et al. [83] and Salgueiro et al. [84]. The method of preparation is also critical, as in the case of the negative results and toxicity detected with spouted bed dried hydroalcoholic extracts of *B. forficata* [133]. The influence of environmental conditions on both the production and the concentration of active compounds should also be considered. Interestingly, kaempferitrin total flavonoid content or flavonoid profiles varied according to the sampling area, altitude and climate in *B. forficata* [146,160] or was influenced by edge-effect in *B. cheilantha* [161]. Adequate botanical identification is also essential. For instance, the subspecies of *B. forficata* are not identified in most assays; thus, flavonoid profiles—and some tested activities—present differences that could be related to variations at the subspecies level or plant misidentification [16,18]. Another variable to be considered is the experimental model selected for conducting the research [140,162]. For example, streptozotocin can induce mild or severe diabetes according to the dose, route of administration or animal strain utilized [163]. Chronic versus acute treatments could also present differences in results, as has been shown above. Thus, it is fundamental to guarantee the quality of the botanical samples, the accuracy of the chemical profiles and the deep research into the action mechanisms before their utilization as phytotherapeutics.

### 3.3. Analgesic Activity

Various studies support the popular therapeutic use of *S. microstachya* for the treatment of pain (Table 1 and Table 2). The methanolic extract (3–30 mg/kg) and the flavonoid quercitrin (1–10 mg/kg) isolated from leaves and administered intraperitoneally, caused potent and dose-related analgesic effects, inhibiting abdominal constrictions induced by an injection of acetic acid in mice (mean ID50 = 7.9 and 2.4 mg/kg, respectively) [39,101]. This extract elicited antinociceptive action against other models of pain such as capsaicin- and formalin-induced licking and was able to reverse, in a dose-related manner, the mechanical hyperalgesia in the rat paw induced by carrageenan, capsaicin, substance P, bradykinin and adrenaline [101]. Furthermore, methanol extract (0.1–2 mg/mL) and ethyl acetate fractions (0.1–2 mg/mL)—enriched in phenols and flavonoids—were found to have antispasmodic activity in vitro, inhibiting the contraction induced by different agonists in smooth muscle preparations of the guineapig ileum and the rat uterus [102].

### 3.4. Anti-inflammatory, Anti-ulcer and Wound Healing Activity

Anti-inflammatory properties mediated by Kunitz proteinase inhibitors isolated from seeds were described in *B. bauhinioides* and tested in animal models (Table 2). This type of inhibitor inhibits blood clotting enzymes, as well as other serine and cysteine proteinases ([164,165,166,167]). In particular, the *B. bauhinioides* cruzipain inhibitor (BbCI) inhibits the enzymes elastase, cathepsin L and cathepsin G [58,59], which are involved in inflammatory processes. Neuhof et al. [49] showed that the pulmonary edema in isolated rabbit lungs caused by activated neutrophils is significantly decreased by BbCI (10^−5^ M). Oliveira et al. [50] proved the effects of the pretreatment of BbCI in rat acute inflammatory models in vivo. BbCI (2.5 mg/kg, intravenous administration, 30 min before carrageenan-induced inflammation) reduced paw edema (24%, 44% and 40% at 2, 3 and 4 h after carrageenan injection, respectively) and the release of the inflammatory mediator bradykinin. It reduced (39%) neutrophil migration into the pleural cavity in a model of pleurisy, as well as the number of rolling, adhered and migrated leucocytes at the spermatic fascia microcirculation in the scrotum. In addition, there was a significant decrease in levels of another mediator, cytokine-induced neutrophil chemo-attractant-1, in the pleural exudate and serum in the inflamed rats. The *B. bauhinioides* kallikrein inhibitor (BbKI) inhibits trypsin, chymotrypsin, plasmin and pancreatic and plasma kallikrein [59,60]. Recombinant rBbKI (2 mg/kg intraperitoneal administration on days 1, 15 and 21) was tested in a model of elastase-induced pulmonary inflammation in mice. Martins-Olivera et al. [53] found that rBbKI treatment attenuated various mechanical alterations of the lung and alveolar septum disruption and reduced the number of inflammatory cells in the bronchoalveolar lavage fluid. In addition, it reduced the cellular expression of several markers of inflammatory recruitment, remodeling the extracellular matrix and oxidative stress responses in airways and alveolar walls, all of which are events involved in the development of chronic obstructive pulmonary disease. Furthermore, rBbCI (2 mg/kg intraperitoneal administration on days 1, 15 and 21) ameliorated the pulmonary mechanics’ changes in C57BL/6 mice elastase-induced pulmonary emphysema, reducing lung tissue destruction, inflammatory alterations, extracellular matrix remodeling and oxidative stress in the alveolar septa and airway walls [52]. 

Lectins isolated from seeds are also involved in anti-inflammatory activities. The *B. bauhinioides* lectin (BBL) was tested in two acute models of inflammation in rats, paw edema and peritonitis. BBL (1 mg/kg intravenously 30 min before carrageenan-induced inflammation) inhibited the paw edema in the second phase (21% and 19% at 3 and 4 h, respectively) [51]. It also inhibited peritoneal neutrophil migration (51% and 64%, when induced by carrageenan and tumor necrosis factor TNF-α, respectively), and decreased leukocyte rolling (58%) and adhesion (68%). The reduction of TNF-α and IL1-β levels would be responsible for anti-inflammatory activity. 

Anti-ulcer activity was reported in *B. holophylla*. Leaf hydroalcoholic extracts enriched in quercetin and myricetin (150 mg/kg oral administration) decreased oxidative stress, attenuated the inflammatory response and favored an antidiarrheal effect in ethanol-induced gastric ulcer in rats [97]. The anti-inflammatory activities were evaluated as the decrease in the production of the pro-inflammatory cytokines TNF-α and interleukin-6 (IL-6) and the increase of the level of the anti-inflammatory cytokine IL-1 [97]. Anti-ulcer activity has also been described in models of acute gastric lesion induced in rats or mice, and aqueous extracts promoted an increase in the amount of gastric mucus [99]. The potential gastroprotective activity is possibly mediated by flavonols. 

Regarding wound healing activities, Rodrigues et al. [119] evaluated ethyl acetate fraction from *B. ungulata* stem bark (FABU 10, 100 μg/mL) using monolayers of human lung adenocarcinoma A549 epithelial cells that were split in the middle. They found that, after 24-h treatment, the cell migration process was accelerated and the initial lesion gap was reduced (32.6–22.0%) compared with the control group. Moreover, they found that a 5-day topical treatment (200 μL of 0.25 or 0.5% *w*/*v* FABU extract gel) significantly reduced a lesion effectuated in the dorsal surface of C57BL/6 mice compared with an untreated control group. Local anti-inflammatory and antioxidant properties were detected, with a reduction of relative expressions of TNFα and IL-1β (50%, FABU at 0.5%) and a reduction of levels of lipid peroxidation (FABU at 0.25% and 0.5%).

### 3.5. Antitumor and Chemoprotective Activity

In the search for natural products for their application in cancer diagnosis or complementary therapy, some promising compounds and extracts have been characterized in six species of *Bauhinia* (Table 2). Moreover, some of them may help to prevent or minimize chemotherapy side effects.

Plant lectins specifically and reversibly bind to different types of carbohydrates or glycoproteins. The alteration of the glycosylation profile of cell surfaces indicates carcinogenesis; lectins have been used in diagnosis or as alternative anticancer drugs [168]. For instance, the glycoprotein *B. forficata* lectin (BfL), purified from *B. forficata* subsp. *forficata* seeds, showed a selective cytotoxic effect (2.5–10 μM) and adhesion inhibition (1 μM) on MCF-7 human breast cancer cells [95]. BfL induced cell death by triggering necrosis and secondary necrosis, with caspase-9 inhibition, and it caused DNA fragmentation, which resulted in cell cycle arrest in the G2/M phase. It also inhibited cell adhesion to laminin, fibronectin and collagen type I, with reduced α1, α6 and β1 integrin subunit expression [95]. Lubkowski et al. [94] evaluated the toxicity of recombinant BfL (1.85 μM) on an NCI-60 panel, which allowed the screening of 60 human cancer cells lines. rBfL showed cytostatic activity and no cytotoxic effects, inhibiting the growth of several cancer cell lines. Inhibition was strong for 5 tumor cell lines (>50%) and moderate for 22 cell lines (10–50%) [94]. In *B. ungulata*, a new galactose-binding lectin—termed BUL—purified from seeds (60–160 µg/mL), showed antiproliferative activity against the HT-29 cell line of human colon adenocarcinoma in a dose-dependent manner [116]. At the most concentrated dose (160 µg/mL), BUL inhibited 80% of cell growth viability. 

Other natural compounds have been isolated from *Bauhinia* plants. Treatment with *B. forficata* HY53 for 24 h inhibited growth in a dose-dependent manner (0.07–0.4 mM, IC50 = 0.13 mM) and induced apoptosis of human hepatocellular carcinoma Hep-G2 cells (apoptotic cell population increased from 8% at 0 mM to 45% at 0.4 mM). Apoptosis would involve activation of caspase-3, a major downstream effector of this process, and then the cleavage of poly(ADP-ribose) polymerase (PARP), critical steps leading to subsequent DNA fragmentation and condensation [91]. In addition, treatment with HY52 for 24 h had an antiproliferative effect (0.07 to 0.41 mM; IC50 = 0.11 mM) and induced apoptosis (at 0.14 mM, the apoptotic cell population increased from 3% at 0 h to 37% at 24 h) on human cervical adenocarcinoma HeLa cells by regulating proteins involved in cell-cycle progression. It induced a G1-phase arrest by inhibiting phosphorylation of retinoblastoma protein pRb via up-regulation of p21WAF1/CIP1 and p27KIP1, and G2/M-phase arrest by downregulation of CDC2 kinase, cyclins A and B1 [92]. Bibenzyl, isolated from the roots of *B. ungulata*, displayed cytotoxicity against pro-myelocytic leukemia (HL-60) and cervical adenocarcinoma (HEP-2) cell lines (IC50 = 4.3 and 6.5 mg/kg, respectively) [118].

Kunitz proteinase inhibitors also mediated effects on cell adhesion and proliferation. Both *B. bauhinioides* BbCI and BbKI reduced HUVEC human umbilical vein endothelial cell proliferation in a concentration-dependent manner [55,56]. Furthermore, compared with chemotherapy cytotoxic drug 5-fluorouracil, recombinant BbCI and rBbKI were more efficient in inhibiting various tumor cell lines [57]. The *B. rufa* trypsin inhibitor (BrTI) and a synthetic peptide containing an RGD motif inhibited cell adhesion to fibronectin of B16F10 and Tm5 murine melanoma cells [109]. In addition, rBbKIm—a recombinant BbKI modified to include the RGD/RGE motifs of the inhibitor BrTI—inhibited the cell viability of prostate cancer cells DU145 and PC3 [108]. In both cancer cell lines, rBbKIm triggered apoptosis and cytochrome c release into the cytosol. rBbKIm caused an arrest at the G0/G1 and G2/M phases and activation of caspase-9 in PC3 cells, whereas, in DU145 cells, the cell cycle was not affected and rBbKIm activated caspase-3 cells. Moreover, it inhibited the in vitro capillary-like tube network formation in HUVECs endothelial cells, which is important to reduce angiogenesis involved in the development of a tumor [108].

Some cytotoxic assays have been developed with the essential oils of *Bauhinia* leaves. *B. ungulata* essential oils exhibited cytotoxic activity against human cancer cell lines HL-60, MCF-7, NCI-H292 and HEP-2, with IC50 ranging from 10.6 µg/mL to 26.6 µg/mL [117]. *B. cheilantha* essential oils also showed in vitro cytotoxic activity against the same human tumor cell lines HL-60, MCF-7, NCI-H292 and HEP-2 (IC50 = 8.6, 18.3, 33.1 and >50 mg/mL, respectively) [61]. 

*Bauhinia* plant extracts or fractions have also shown antitumor and/or chemoprotective effects. For instance, *B. ungulata* extracts of stems, enriched in flavonoids and alkaloids, inhibited the activity of matrix metalloproteinases MMP-2 and MMP-9, which cleave the main structural components of the basal membrane and have a prognostic influence on human cancers [121]. More recently, Ribeiro et al. [98] found that *B. holophylla* hydroalcoholic extract induced apoptosis and showed high antiproliferative effects in Hep-G2 cells. The extract did not induce mutagenicity at three concentrations tested and had protective effects against DNA damage produced by carcinogenic agents such as benzo[a]pyrene (B[a]P). Aqueous extracts of *B. forficata* have antimutagenic/protective action on bone marrow cells of Wistar rats; they reduced chromosomal alterations induced by the chemotherapeutic agent cyclophosphamide [96]. *B. forficata* flavonoid-rich fraction and purified kaempferitrin protected intestinal cells (IEC-6 cells) from cytotoxicity induced by irinotecan [93]. This chemotherapy agent—used to treat colorectal cancer—produces side effects such as damage in intestinal mucosa and mucositis. The flavonoid-rich fraction (100 mg/kg/day oral administration for 14 days) prevented mucositis in mice (attenuating diarrhea and histological damage in the duodenum and the colon, among other tested parameters), without interfering in irinotecan antitumor activity. Furthermore, this fraction produced a significant antitumoral effect on a murine melanoma model.

### 3.6. Antimicrobial Activity

Concerning antimicrobial activity, Alves et al. [70] showed that *B. forficata* leaf ethanolic extracts had antimicrobial activity against *Candida albicans*. Sousa et al. [73] did not detect activity against *C. albicans*, *Escherichia coli* or *Staphylococcus aureus* species, but the extracts increased the effectiveness of norfloxacin against the S. aureus SA1199-B with a concentration-dependent effect. This strain overproduces the NorA efflux pump, a transmembrane protein that extrudes antimicrobial compounds, such as norfloxacin. Thus, *B. forficata* extract could be potentially used—together with norfloxacin—to treat infections caused by multidrug-resistant *S. aureus* [73]. Ferreira-Filho et al. [71,72] showed antimicrobial effects of *B. forficata* leaf tincture (20% in a 70% hydroethanolic solution) against oral microorganism strains and mature dental biofilms obtained from salivary samples and formed on membranes or bovine enamel blocks. The tincture is a promising preventive agent of dental caries, with no cytotoxic effect, tested against oral fibroblast cells. Miceli et al. [35], however, did not detect an antimicrobial effect against different strains of bacteria and yeasts, nor did Simões and Almeida [169] with an ethanolic extract of stem bark against Klebsiella pneumoniae*, E. coli* and S*. aureus.* Aqueous and ethanolic extracts of *B. rufa* presented antimicrobial activity against *Candida spp.* [107] and *B. ungulata* aqueous and esential oils presented antimicrobial activity against various pathogenic microorganisms alone (Medeiros et al. [113] or in synergy with antibiotics [112].

### 3.7. Anticoagulant, Antithrombotic, Antihypertensive and Diuretic Activity

Since the Kunitz proteinase inhibitor BbKI form *B. bauhinioides* is active against enzymes involved in coagulation processes, fibrinolysis and inflammation, Brito et al. [54] evaluated its antithrombotic activity in vein and arterial thrombosis models in rat and mice, respectively. They found that BbKI (2.0 mg/kg) reduced the venous thrombus weight by 65% and prolonged the time for total artery occlusion (87.27 ± 14.94 min) in comparison with animals in the control groups (51.97 ± 10.52 min); these results indicated thrombosis prevention. The lectine BfL—from *B. forficata* subsp. *forficata*—exhibited anticoagulant and antiplatelet aggregating properties in biological models of homeostasis in vitro [64]. Purified BfL (1.5–4 µM) increased coagulation time (an effect not related to human plasma kallikrein or human factor Xa inhibition) and inhibited ADP and epinephrine-induced platelet aggregation in a dose-dependent manner. *B. rufa* hexane extracts of leaves produced 26.11% of clot lysis from human venous blood [110].

Regarding vasorelaxant properties, it has been demonstrated that aqueous–ethanol extracts of leaves of *B. candicans* (120 mg/kg/day for 2 weeks) increased the endothelium-dependent relaxation of phenylephrine-precontracted aortic rings in ALX diabetic rats; this effect was attributed to the antioxidant activity mediated by flavonoids [68]. Acetylcholine-induced relaxation of aortic rings was greater in diabetic rats treated with extracts than in untreated diabetic rats. The vasorelaxant properties of ethyl acetate plus butanol fraction from *B. forficata* leaves (1–50 μg/mL) were also described in aortic rings of both normotensive and hypertensive rats precontracted with phenylephrine [69]. The effect was found in aorta rings with intact endothelium and endothelium-denuded aorta. The modulation of vascular tone would be related with the nitric oxide/soluble guanylate cyclase pathway, since the incubation with a non-selective nitric oxide synthase inhibitor (L-NAME) or a soluble guanylate cyclase inhibitor (ODQ) blocked the vasorelaxant activities of the extract. Potassium channels and membrane hyperpolarization would also be involved in vascular tone. The flavonoids kaempferitrin and kaempferol (0.001–0.3 μg/mL) showed a vasorelaxant potential of 34.70% and 40.54%, respectively. Anjos et al. [66] found that the aqueous extract of *B. forficata* (5–40 mg/kg intravenous administration) presented antihypertensive effects, inducing a dose-dependent transitory hypotension and tachycardia in normotensive rats and reducing mean arterial pressure by 12% in hypertensive rats (oral acute dose of 400 mg/kg). These effects seem to involve the release of nitric oxide. 

*Bauhinia forficata* is popularly consumed for kidney and urinary disorders such as polyuria, cystitis and kidney stones (Table 1). Debenedetti et al. [170] could not demonstrate diuretic properties with plant infusions in rats (250, 500 and 1000 mg/kg oral administration). Afterwards, Toloza-Zambrano et al. [138] reported an increase in diuresis in human diabetic patients consuming leaf infusions (see Section 3.1). More recently, Souza et al. [67] have reported diuretic and natriuretic properties of leaf extracts. When orally treated with leaf aqueous infusion (300 mg/kg) and other fractions, urine volume and electrolyte levels significantly increased after 8 h in both normotensive and spontaneously hypertensive rats compared with controls, with no changes in pH, density or conductivity parameters. Moreover, isolated kaempferitrin (0.3 and 1 mg/kg) induced diuresis and saluresis and augmented excretion of urinary creatinine and prostaglandin E2. Diuretic action should be related with the generation of prostanoids, since this activity is affected by treatment with the cyclooxygenase inhibitor indomethacin. Further, it was demonstrated that afzelin—a flavonoid from the kaempferitrin metabolic route—but not kaempferol, presents acute and prolonged diuretic action and renal protective action; diuresis should involve endogenous prostanoid generation and muscarinic receptor activation [171]. 

Thus, these plants could play an interesting role as alternative pharmacotherapies in renal or cardiovascular disorders [69,171]. Moreover, endothelial dysfunction [68] or hypertension [66] are sometimes associated with diabetes.

### 3.8. Other Biological Activities

Other diverse biological activities have been described in austral South American *Bauhinia* species (Table 2). The chronic use of antipsychotics can trigger adverse motor effects such as the repetitive involuntary movements seen in tardive dyskinesia in humans. Since these disturbances seem to be related to oxidative stress in some areas of the brain, Peroza et al. [80] investigated the effects of *B. forficata* on brain lipid peroxidation in a model of orofacial dyskinesia in rats induced by long-term treatment with the antipsychotic haloperidol (38 mg/kg every 28 days). Plant decoction (250–300 mg/kg/day for 16 weeks) prevented the formation of lipid peroxidation induced by two pro-oxidants tested. Moreover, it partially diminished the vacuous chewing movements induced by haloperidol. 

The *B. bauhinioides* BbCI inhibition of cruzipain—a cysteine proteinase from Trypanosoma cruzi—has shown the potential of this species for the development of anti-Chagas drugs [58,59]. Santos et al. [100] reported that hydroethanolic extracts of *B. holophylla*, enriched in flavonoids, presented a potent activity (IC50 = 3.2 µg/mL, selectivity index = 27.6) against the Dengue virus serotype DENV-2, which is transmitted by mosquitoes. Leaf essential oils of *B. cheilantha* and *B. ungulata* showed larvicidal potential against instar III larvae of the *Aedes aegypti* mosquito (LC50 = 40.84 ± 0.87 mg/mL; LC50 = 75.1 ± 2.8 µg/mL, respectively) [61,117]. Other *Bauhinia* extracts presented larvicidal activity against *A. aegypti* (*B. cheilantha*, woods and seeds) and *Culex quinquefasciatus* (*B. rufa*, leaves) [62,63,111]). 

Hydromethanolic extracts of leaves of both subspecies of *B. forficata* exhibited some activity against cholinesterases (acetyl- or butyrylcholinesterase) [78], whereas hexane extracts of flowers and leaf essential oils of *B. ungulata* inhibited acetylcholinesterase [113,120]. These enzymes are associated with the etiology of Alzheimer’s disease; therefore, these species may potentially contribute to the treatment of this pathology [78]. 

Oliveira et al. [65]) found that the aqueous extract of the aerial parts of *B. forficata* is a promising source of natural inhibitors of the serine proteases involved in blood clotting disturbances induced by snake venoms. The extract neutralized the clotting activity induced by the *Bothrops* and *Crotalus* crude venoms and inhibited clotting and fibrinogenolytic activities induced by the isolated thrombin-like enzyme from the *Bo. jararacussu* venom. It also inhibited the edema induced by *C. durissus terrificus* venom in mice. On the other side, *B. forficata* extracts enhanced the *Tityus serrulatus* scorpion venom’s lethality [130].

Finally, the presence of lectins and Kunitz inhibitor activities could have potential uses that still have not been explored [164,165,166,168]. For instance, Castro et al. [172] have produced and characterized a lectin from the primary callus cultures of *B. holophylla*. Other proteinase inhibitors (BuXI) and their target proteinases have been characterized in *B. ungulata* [122] and two isoforms of Kunitz-type trypsin inhibitor-like 1(BrTI and α-chain) were identified in a *B. forficata* proteome [156].

### 3.9. Toxicity and Adverse Effects

Toxic effects were not reported in most methodological approaches (e.g., [72,76,84,89,97,138,141,173]), however some research findings deserve attention. It has been reported that acute treatment with *B. forficata* crude extract (2.85 g/kg) injected intraperitoneally caused the death of 50% of the animals, yet oral administration (0.5 to 5.0 g/kg) is not toxic [1]. The increment in hepatic toxicity markers triggered by *B. forficata* spouted bed-dried extract and *B. holophyla* aqueous extracts in treated diabetic rats suggests liver injury (see Section 3.1) [133,140]. In the first case, this could be attributed to secondary product formation or interaction with Tixosil employed in the experiments. Low toxicity was reported for *B. forficata* stem bark ethanolic extracts in *Artemia salina* tests (CL50 = 853.80 μg/mL) and the authors recommended a dilution when preparing formulations, teas and garrafadas [169]. Cavalcanti et al. [174] warned about toxicologic effects of *B. forficata* aqueous extracts (5 g/kg oral administration), since they detected alterations in behavior in rat anxiolytic models, such as decreased general activity and increased grooming duration and pentobarbital sleep inducing time; these authors suggest a dosage for central neurotransmitters. Sampaio et al. [88] reported damage in epididymal tissues in rats treated with *B. forficata* extracts (0.1 mL/10 g body weight/day alcoholic extract for 30 days). Mitochondrial damage was also reported by Ecker et al. [82] in isolated rat liver mitochondria exposed to *B. forficata* aqueous extracts in vitro. They detected a decrease in mitochondrial dehydrogenase activity at high concentrations of extracts (200 and 400 μg/mL) and an induction of swelling (at 25 and 400 μg/mL). Finally, by interviewing 100 *Bauhinia* spp. consumers in Diadema, Sau Paulo (Brazil), Neto et al. [175] reported adverse reactions in two persons (mother and daughter) who presented a strong allergic reaction after the consumption of tea and had to be hospitalized; the causes were unclassifiable by the authors. They informed of four records of severe reactions after consumption of an unregistered medication of *B. forficata* (hepatic problems, such as cirrhosis and renal pain), published by the Brazilian sanitary vigilance agency; they did not find similar reports in the literature searches. Thus, it is important to further investigate the possible adverse effects of the consumption of the *Bauhinia* species in order to minimize the health risk.

## 4. Conclusions

*Bauhinia forficata*, *B. ungulata*, *B. bauhinioides*, *S. microstachya*, *B. holophylla*, *B. rufa* and *B. cheilantha* are the austral South American species with records of pharmacological properties that explain their various ethnopharmacological uses. *Bauhinia forficata* is the most consumed and studied plant, with antidiabetic, antioxidant, anticoagulant, antihypertensive, diuretic, antimicrobial and antitumor properties. Together with *B. holophylla*, they are important for their antidiabetic properties, since several studies indicate their effectiveness as a hypoglycemic agent. Conflicting results could be explained by differences in extraction methods and preparation, chemical profiles, route of administration and dose, treatment periods, animal models used or plant identification. Clinical studies in *B. forficata* are still preliminary and deserve further investigation. *B. bauhinioides* is distinguished for its anti-inflammatory and antithrombotic activities mediated by Kunitz-type inhibitors. *S. microstachya* is distinguished for its analgesic properties. Anti-ulcer and wound healing activities recorded in *B. holophylla* and *B. ungulata*, respectively, are of particular interest. Most of the species possess antitumor activity, mediated by lectins, Kunitz proteinase inhibitors and other compounds. *B. forficata* extracts alleviate the side effects of chemotherapy, such as intestinal mucositis. The antioxidant capacity of flavonoids and other bioactive compounds present in *B. forficata*, *S. microstachya*, *B. ungulata* and *B. holophylla* make these plants good candidates for assisting or treating various alterations related with oxidative stress, such as diabetic complications and gastric ulcer, or even for cosmetic use. Thus, these regional species constitute promising targets for new bioactive substance research and phytotherapy.

## Figures and Tables

**Table 1:** Main traditional uses of austral South American *Bauhinia*.

Species		Traditional Uses	References
*affinis*		Diabetes.	[7,24]
*argentinensis* *		Analgesic (kidney). Hepatic disorders. Kidney disorders.	[8,25]
*bauhinioides*		Diuretic. Kidney disorders. Refrigerant.	[8,26]
*cheilantha*		Analgesic (back, pain in general, headache). Anemia. Anti-inflammatory. Antilipidemic. Asthma. Blood thinner.Cancer. Depurative. Diabetes. Digestive disorders. Dysphonia and throat inflammation. Flu and cough, expectorant. Hemostatic. Helminthiasis. Hypertension. Hypocholesterolemic agent. Inappetence. Kidney disorders. Rheumatism. Sedative. Sexual impotence. Tonic. Triglyceride reducer. Urinary infection, burning in the urethra, uterus.	[3,27,28,29,30]
forficata **		Antiseptic. Cardiovascular disorders. Diabetes. Hypoglycemic agent. Diuretic. Endocrine disorders. Gastrointestinal disorders. Gynecologic and obstetrics disorders. Hepatic disorders. Hypocholesterolemic agent. Indigestion flatulence. Kidney disorders. Urinary disorders. Weakness.	[9,13,14,24,26,29,31,32,33,34,35]
	*p*	Abluent. Analgesic (headache). Antidandruff. Antihemorrhoidal. Antinephritic. Antitussive expectorant. Astringent. Blood depurative. Cardiotonic. Diabetes. Hypoglycemic agent. Digestive. Diuretic. Genito-urinary and hemolymphatic system. Hypotensive agent. Rheumatism. Vulnerary.	[8,10,25,36,37,38]
	*c*	Cistitis. Diabetes. Hypoglycemic agent. Kidney disorders, kidney stones.	[1,2,3]
*holophylla*		Anti-obesity. Astringent. Diabetes. Hypoglycemic agent. Diuretic.	[3]
*microstachya*		Analgesic. Anti-inflammatory. Blood depurative. Diabetes. Hypoglycemic agent. Liver pain, spleen ache. Respiratory disorders. Urinary and gallbladder disorders.	[8,13,15,31,37,39,40]
*mollis*		nd.	[41,42]
*rufa*		Anoretic. Antihyperlipidemic agent. Astringent. Diabetes. Hypoglycemic agent. Diuretic.	[3,41,43,44]
*ungulata*		Analgesic (stomach). Diabetes. Hypoglycemic agent. Hypocholesterolemic agent. Hypolipidemic agent. Laxative.	[3,29,41,45,46]

* var. *argentinensis*. ** Nomenclature according to published papers. *c*—*B. candicans*; *p*—*B. forficata* subsp. *pruinosa*; nd—author/s mentioned the species as a medicinal species without specifying the type of use.

**Table 2:** Biological activity of austral South American *Bauhinia*. The activities are grouped into eight categories (see Figure 2 and Section 3.1, Section 3.2, Section 3.3, Section 3.4, Section 3.5, Section 3.6, Section 3.7 and Section 3.8 in the text). *bau—bauhinioides*; *che—cheilantha*; *for—forficata*; *hol—holophylla*; *mic—microstachya*; *ruf—rufa*; *ung—ungulata*.

Species	Biological Activity		Study Type	Extract/ Compound	Part Used	Study Model/Target Species/Cells/Enzymes/Method Investigated	References
	Category Detail						
*bau*	Anti-inflammatory		VIV	BbCI	seed	Rabbit-activated neutrophil-induced pulmonary edema.	[49]
			VIV	BbCI	seed	Rat carrageenan-induced paw edema and pleurisy. Scrotal microvasculature.	[50]
			VIV	BBL	seed	Rat carrageenan-induced paw edema and carrageenan or TNF-α-induced peritonitis.	[51]
			VIV	rBbCI		Mice elastase-induced pulmonary emphysema.	[52]
			VIV	rBbKI		Mice elastase-induced pulmonary inflammation.	[53]
	Antithrombotic		VIV	BbKI	seed	Vein and arterial thrombosis models in rats and mice.	[54]
	Antitumor	Antiproliferative activity.	VIT	BbCI, BbKI	seed	HUVEC human umbilical vein endothelial cells.	[55,56]
			VIT	rBbCI, rBbKI		MKN-28 Hs746T (gastric), HCT116 HT29 (colorectal), SkBr-3 MCF-7 (breast), THP-1 and K562 (leukemia) human cancer cells.	[57]
	Other biological activities	Kunitz-tipe proteinase inhibitors activity.	VIT	BbCI	seed	Elastase cathepsin L and cathepsin G.	[58,59]
			VIT	BbKI	seed	Trypsin chymotrypsin plasmin and pancreatic and plasma kallikrein.	[59,60]
		*Trypanosoma cruzi* cruzipain inhibitor activity.	VIT	BbCI	seed	*Trypanosoma cruzi* cruzipain.	[58,59]
*che*	Antitumor	Cytotoxic activity.	VIT	Essential oils	leaf	HL-60 (leukemia), MCF-7 (breast), NCI-H292 (lung) and HEP-2 (endocervical) human cancer cells.	[61]
	Other biological activities	Insecticide.	VIT	Crude ext	seed	*Aedes aegypti.*	[62]
		Larvicide.	VIT	Ethanolic ext	wood	*Aedes aegypti.*	[63]
			VIT	Essential oils	leaf	*Aedes aegypti.*	[61]
	Anticoagulant, antihypertensive and diuretic	Anticoagulant and antiplatelet aggregating properties.	VIT	BfL	seed	Biological models of homeostasis. Human blood samples.	[64]
		Anticoagulant, antifrinogenolytic activities (snake venoms).	VIT	Aqueous ext	leaf	Clotting disturbances in human blood samples induced by snake venoms.	[65]
		Antihypertensive effects.	VIV	Aqueous ext	leaf	Normotensive and hypertensive rats.	[66]
		Diuretic and natriuretic activity.	VIV	Aqueous infusion, methanolic ext, trichloromethane, ethyl acetate-butanolic fra, kaempferitrin	leaf	Normotensive and spontaneously hypertensive rats.	[67]
		Vasorelaxant properties.	VIV *c*	Hydroethanolic ext	leaf	Aortic rings of alloxan-induced diabetic rats.	[68]
			VIV	Ext and fra, kaempferitrin, kaempferol	leaf	Aortic rings of normotensive and hypertensive rats.	[69]
	Antimicrobial		VIT	Hydroethanolic ext	leaf	*Candida albicans.*	[70]
			VIT	Hydroethanolic solution	leaf	Oral microorganism strains and mature dental biofilms.	[71,72]
			VIT	Ethanolic ext	leaf	*Staphylococcus aureus* SA1199-B.	[73]
	Antioxidant		VIT	Kaempferitrin	leaf	Peroxidation induced by ascorbyl radical in microsomes or in asolectin and phosphatidylcholine liposomes. DPPH assays and MPO activity.	[74]
			VIT	Aqueous ext	leaf	Superoxide anion radical scavenging MPO activity and ABTS radical cation assay.	[75]
			VIV	Aqueous ext	leaf	Pregnant streptozotocin-induced diabetic rats blood. DNTB assay.	[76]
			VIT	Spray and spouted bed dried ext	leaf	DPPH and lipid peroxidation assay.	[77]
			VIT *f,p*	Hydromethanolic ext	leaf	DPPH assay NO and superoxide radicals scavenging activity.	[78]
			VIT	Methanolic ext and fra	leaf	DPPH assay.	[79]
			VIT VIV	Decoction	leaf	Lipid peroxidation (TBARS). Orofacial dyskinesia induced by long-term treatment with haloperidol in rats.	[80]
			VIT *p*	Aqueous infusion	leaf	Human erythrocytes exposed to high glucose concentrations and egg yolk samples. Non-protein SH levels and lipid peroxidation (TBARS assay). Iron chelating DPPH and deoxyribose degradation assays.	[81]
			VIT	Aqueous ext	leaf	DPPH assay. Fe2+/citrate-mediated mitochondrial lipid peroxidation in isolated rat liver mitochondria.	[82]
			VIT	Hydromethanolic ext	leaf	DPPH assay.	[83]
			VIT	Hydroalcoholic ext, flavonoid-rich fra	leaf	DPPH assay measurement of reducing power and ferrous ions chelating activity.	[35]
			VIV *p*	Aqueous infusion	leaf	Streptozotocin-induced diabetic mice pancreas. Lipid peroxidation assay (TBARS) DCFH oxidation assay and NADPH quinone oxidoreductase 1 expression levels.	[84]
			VIT	Aqueous infusion	leaf	*Drosophila melanogaster* fed on high-sucrose diet.	[85]
			VIT	Ethanolic and hexane ext	leaf	DPP and ORAC assays.	[86]
			VIV	Commercial Ethanolic ext	leaf	Liver from rats exposed to Bisphenol A. TBARS assay.	[87]
			VIV	Ethanolic ext	leaf	Rat male genital system. TBARS assay.	[88]
			VIT	Ethanolic ext fra	leaf	DPPH ORAC and FRAP assays.	[89]
	Antitumor and chemoprotective	Antiproliferative activity.	VIT	BfL-II rBfL-II	seed	HT-29 (colon) and MCF-7 (breast) human cancer cells.	[90]
		Antiproliferative and apoptotic activities.	VIT	HY53	leaf	Hep-G2 (liver) human cancer cells.	[91]
				HY52	leaf	HeLa (cervical) human cancer cells.	[92]
		Antitumoral activity.	VIV	Flavonoid-rich fra, purified kaempferitrin	leaf	Murine melanoma.	[93]
		Cytostatic activity.	VIT	rBfL		Several cancer cell lines (e.g., melanoma non-small cell lung ovarian renal and breast) included in the NCI-60 panel.	[94]
			VIT	Hydroethanolic ext	leaf	MCF-7(breast), NCI-ADR/RES (ovary with phenotype resistance to multiple drugs), 786-O (kidney), NCI-H460 (lung), OVCAR-3 (ovary), HT-29 (colon), K562 (bone marrow) human cancer cells and HaCaT (normal keratinocyte) cell line.	[70]
		Cytotoxic activity.	VIT	BfL	seed	MCF-7 (breast) human cancer cells.	[95]
			VIT	Hydro-alcoholic ext	leaf	FO-1 (melanoma) human cells.	[35]
		Chemoprotective effects.	VIT	Aqueous ext	leaf	Bone marrow cells of Wistar rats exposed to clophosphamide.	[96]
			VIT VIV	Flavonoid-rich fra, kaempferitrin	leaf	Intestinal cells (IEC-6 cells) exposed to irinotecan. Irinotecan-induced mucositis in mice.	[93]
	Other biological activities	Edema inhibition (induced by snake venoms).	VIV	Aqueous ext	leaf	Edema induced by *Crotalus durissus terrificus* venom in mice.	[65]
		Inhibition of cholinesterase activity.	VIT *f,p*	Hydromethanolic ext	leaf	Cholinesterases (acetyl- or butyrylcholinesterase).	[78]
		Protection against vacuous chewing movements induced by haloperidol.	VIV	Decoction	leaf	Orofacial dyskinesia induced by long-term treatment with haloperidol in rats.	[80]
*hol*	Antioxidant		VIV	Hydroalcoholic ext enriched in quercetin and myricetin.	leaf	Ethanol-induced gastric ulcer in rats.	[97]
	Antitumor	Antiproliferative and apoptotic activity. Protective effects against DNA damage.	VIT	Hydroalcoholic ext enriched in isorhamentin and quercetin derivatives.	leaf	Hep-G2 (liver) human cancer cells.	[98]
	Anti-ulcer	Anti-ulcer activity. Antidiarrheal effect.	VIV	Hydroalcoholic ext enriched in quercetin and myricetin.	leaf	Ethanol-induced gastric ulcer in rats.	[97]
		Anti-ulcer activity.		Aqueous ext	leaf	HCl-Ethanol-induced gastric ulcer in rats or mice. NSAIDS-Bethanecol induced gastric ulcer in mice.	[99]
	Other biological activities	Anti-dengue activity.	VIT	Hydroethanolic ext	leaf	Dengue virus serotype DENV-2.	[100]
*mic*	Analgesic	Analgesic.	VIV	Methanolic ext Quercitrin	leaf	Abdominal constrictions induced by injection of acetic acid in mice and capsaicin- and formalin-induced licking.	[39,101]
		Antihyperalgesic.	VIV	Methanolic ext Quercitrin	leaf	Carrageenan- capsaicin- substance P- bradykinin- and adrenaline-induced mechanical hyperalgesia in rat paw.	[101]
		Antispasmodic effect.	VIV	Methanol ext, ethyl acetate fra	leaf	Smooth muscle preparations of guineapig ileum and rat uterus.	[102]
	Antioxidant		VIT	Hydroalcoholic ext	leaf	DPPH and phosphomolybdenum assays.	[103]
			VIT	Aqueous and hydroethanolic ext	leaf	TRAP TEAC TBARS NO superoxide and hydroxyl radical assays.	[15]
			VIT	Various ext and fra	leaf, stem	DPPH and phosphomolybdenum assays.	[104]
			VIT *m*	Ethnolic ext and fra	leaf	DPPH ORAC and ABTS assays.	[105]
	Other biological activities	Photoprotective.	VIT/ HUM	Oil-in-water emulsions/sunscreens and water-acetone or activated carbon treated-ethanol ext	leaf	In vitro sun protection factor determination and UVA protection factor assessment. Colipa test in human volunteers to assess sun protection factor.	[106]
*ruf*	Antimicrobial		VIT	Aqueous and ethanolic ext	leaf	Strains of *Candida spp.*	[107]
	Antitumor	Apoptotic activity.	VIT	rBbKIm, modified with RGD/RGE motifs of BrTI		DU145 and PC3 (prostate) human cancer cells.	[108]
		Inhibition of adhesion.	VIT	BrTI and synthetic peptide containing RGD motif	seed	B16F10 and Tm5 murine melanoma cells.	[109]
		Inhibition of capillary-like tube network formation.	VIT	rBbKIm, modified with RGD/RGE motifs of BrTI		HUVEC human umbilical vein endothelial cells.	[108]
	Thrombolytic activity.		VIT	Hexane ext	leaf	Human venous blood samples. Clot lysis.	[110]
	Other biological activities	Kunitz-tipe proteinase inhibitors activity.	VIT	BrTI	seed	Plasma kallikrein and trypsin.	[109]
		Larvicide.	VIT	Metanolic ext and fra. Butane, hexane, dichloromethane, ethyl acetate	leaf	*Culex quinquefasciatus.*	[111]
*Ung*	Antimicrobial		VIT	Aqueous	leaf	*Staphylococcus aureus Escherichia coli* and *Pseudomonas aeruginosa.*	[112]
			VIT	Essential oils	leaf	*Candida albicans Bacillus cereus Salmonella typhimurium Staphylococcus aureus* and *Citrobacter freundii.*	[113]
	Antioxidant		VIT	Ethanolic ext and fra (chloroform, ethyl acetate, hexane, hydroalcoholic)	leaf	DPPH phosphomolybdenum and lipid peroxidation (TBARS) assays.	[114]
			VIT	Ethyl acetate fra	stem	Phosphomolibdenum ROS NO hydrogen peroxide and lipid peroxidation (TBARS) assays in LPS- RAW 264.7 stimulated macrophages.	[115]
	Antitumor	Antiproliferative activity.	VIT	BUL	seed	HT-29 (colon) human cancer cells.	[116]
		Cytotoxic activity.	VIT	Essential oils	leaf	HL-60 (leukemia), MCF-7 (breast), NCI-H292 (lung) and HEP-2 (cervical) human cancer cells.	[117]
			VIT	Bibenzyl	root	HL-60 (leukemia) and Hep-2 (cervical) human cancer cells.	[118]
	Wound healing		VIV	Ethyl acetate fra	leaf	Surgical wound model in mice. Monolayers of A549 (lung adenocarcinoma) human epithelial cells.	[119]
	Other biological activities	Inhibition of acetylcholinesterase activity.	VIT	Hexane ext	flower	Acetylcholinesterase.	[120]
			VIT	Essential oils	leaf	Acetylcholinesterase.	[113]
		Inhibition of matrix metalloproteinases activity.	VIT	Ethyl acetate partition	stem	Matrix metalloproteinases MMP-2 and MMP-9.	[121]
		Kunitz-tipe proteinase inhibitors activity.	VIT	BuXI	seed	Trypsin and kallikrein.	[122]
		Larvicide.	VIT	Essential oils	leaf	*Aedes aegypti.*	[117]

Nomenclature according to published papers: *f*—*B. forficata* subsp. *forficata*; *p—B. forficata* subsp. *pruinosa*; *c—B. candicans*; *m—B. microstachya* var. *massambabensis*. ABTS—2,2′-azino-bis (3-ethylbenzothiazoline-6-sulfonic acid; BbCI—*B. bauhinioides* cruzipain inhibitor; BbKI—*B. bauhinioides* kallikrein inhibitor; BBL—*B. bauhinioides* lectin; BfL—*B. forficata* lectin; BfL-II—*B. forficata* lectine II; BrTI—*B. rufa* trypsin inhibitor; BUL*—B. ungulata* lectine; BuXI—*B. ungulata* factor Xa inhibitor; DCFH—2,7-dchlorofluorescein; DNTB—5,5-dithio-bis(2-nitrobenzoic acid); DPPH—2,2-diphenyl-1-picrylhydrazyl; ext—extract; fra—fraction; FRAP—ferric reducing ability of plasma; HUM—human study; MPO—myeloperoxidase; NO—nitric oxide; ORAC—oxygen radical absorbance capacity; r—recombinant; ROS—reactive oxygen species; TBARS—thiobarbituric acid reactive substances; TEAC—Trolox equivalent antioxidant capacity; TNF-α—tumor necrosis factor α; TRAP—total radical-trapping antioxidant parameter; VIT—in vitro study; VIV—in vivo study.

**Table 3:** Main antidiabetic properties and related activities of *Bauhinia forficata* and *B. holophylla*. Model animals were Wistar rats, Swiss mice or New Zealand rabbits, except where indicated.

Spp	Study Type/Treatment/ Time	Extract/ Compound	Doses/Day Tested	Study Model/Enzymes/ Method	Gender	Basal Glycemia	Effects	References
for *	VIV/ O/40d *c*	Aqueous infusion	20 g/L	ALX diabetic rats.	M	X = 181 and 36,927 mg/dL	– No hypoglycemic activity. – No improvement in glucose tolerance. – No reduction in cholesterol levels. – No reduction in water and food intake. – No changes in body weight.	[123]
	VIV/O/31d	Decoction	150 g leaf/L; 352 ± 78 mL/kg	STZ diabetic rats and normal rats.	M	>500 mg/dL	+ Hypoglycemic activity in diabetic rats. – No hypoglycemic activity in normal rats. + Reduction in urine glucose levels. – No changes in hepatic glycogen. – No reduction in triglycerides and cholesterol. + Reduction in urinary urea. – No reduction in food and liquid intake. – No changes in body weight. – No reduction in urinary volume.	[124]
	VIV/O/A	N-butanol fra	400, 600, 800 mg/kg	ALX diabetic rats and normal rats.	M	X = 3305 mg/dL	+ Hypoglycemic activity in diabetic and normal rats. – No improvement in glucose tolerance in normal rats.	[125]
	VIV/O/20d	Aqueous ext	500–1000 mg/kg	STZ diabetic pregnant rats	F	>200 mg/dL	+ Increment of hepatic glycogen. – No hypoglycemic activity. – No control of total lipid, triglyceride and cholesterol levels (lower mean values observed). + Reduction in uric acid concentration. – No changes in total protein and albumin levels.	[126]
	VIV O/A IV/A *c*	Methanolic ext and fra butanolic fra	8 mg/kg	ALX diabetic rabbits.	F-M	250–320 mg/dL	+ Hypoglycemic activity. + Improvement in glucose tolerance. + Reduction in urine glucose levels. + Reduction in urine volume.	[127]
	VIV/ O/A VIT	Purified kaempferitrin	100 mg/kg	ALX diabetic rats and normal rats. Soleus muscle from diabetic and normal rats.	M	25–30 mmol/l	+ Hypoglycemic activity in diabetic rats. + Stimulatory effect of glucose uptake in muscle from normal rats. – No reduction in glucosuria in normal and diabetic rats. – No changes in protein synthesis in muscle from normal and diabetic rats.	[128]
	VIV/O/7d	Aqueous, ethanolic and hexanic ext	200 and 400 mg/kg	ALX diabetic rats.	M	>200 mg/dL	+ Hypoglycemic activity. + Reduction in triglycerides, total cholesterol and HDL-cholesterol. – No reduction in LDL levels.	[129]
	VIV/O/A	Purified kaempferitrin	50, 100, 200 mg/kg	ALX diabetic rats and normal rats.	M	25–30 mmol/l	+ Hypoglycemic activity in normal and diabetic rats. – No improvement in glucose tolerance in normal rats.	[74]
	VIV/O/A	Aqueous infusion	1 g/kg/0.5 mL wat er	Rats and mice exposed to *Tityus serrulatus* scorpion venom.	M		+ Hypoglycemic activity in treated rats. – No hypoglycemic activity in untreated rats. + Delay in glycogenolysis (but not avoidance). – Decrease of serum levels of insulin induced by venom. – Enhanced venom lethality in mice.	[130]
	VIV/O/A	Aqueous ext	10% *w*/*v*	Normoglycemic mice.	M		+ Hypoglycemic activity.	[131]
	VIV/O/20d	Aqueous ext	500–1000 mg/kg, 2 doses	STZ diabetic pregnant rats and normal rats.	F	>300 mg/dL	– No hypoglycemic activity in diabetic and normal rats. – No improvement of various maternal reproductive outcomes in diabetic rats.	[76]
	VIV/O/7d	Spray-dried, oven-dried, wet granulated ext	200 mg/kg	STZ diabetic rats.	M	>200 mg/kg body wt	+ Hypoglycemic activity. – No prevention of decrease in liver glycogen.	[132]
	VIV/ O/14d *c*	Aqueous-ethanol ext	120 mg/kg	ALX diabetic rats.	F-M	200–300 mg/dL	+ Hypoglycemic activity.	[68]
	VIV/O/35d	Spouted bed-dried hydroalcoholic ext	0.125 g/L and 0.25 g/L, 2 doses of 1 mL	STZ diabetic rats.	M	X = 514 mg/dL	– No hypoglycemic activity. – No reduction in urinary glucose. – No reduction in cholesterol, triglycerides and HDL-cholesterol levels. – No reduction in water and food intake. – No changes in body weight. – No reduction in urine volume, urinary urea and proteinuria. – Hepatic toxicity: increament of aspartate and alanine aminotransferase activities.	[133]
	VIV/O/20d	Aqueous ext	800 mg/kg	Nonobese diabetic (NOD) mice. Isolated salivary glands.	F	>300 mg/dL	+ Hypoglycemic activity. + Weight recovery. + Reduction in urine pH and proteinuria. – No improvement in salivary glands tissue recovery.	[134]
	VIV/ O/21d *p*	Aqueous infusion	1 mg/mL (313 mg/kg)	STZ diabetic mice.	M	>300 mg/dL	– No hypoglycemic activity. – No changes in liver/body weight ratio.	[84]
	VIV	Aqueous infusion	5 mg/mL medium	*Drosophila melanogaster* fed on high-sucrose diet.	-		+ Reduction of hemolymph glucose levels. + Reduction of the hemolymph levels of triacylglycerols. + Improvement of the effects induced by diet intake (developmental time, survival, body weight).	[85]
	VIT *c*	Butanol ext	0.001–0.07 mg/mg protein	Isolated gastric glands of ALX diabetic and normal rabbits.	F-M	200–260 mg/dL	+ Stimulatory effect of glucose uptake in normal and diabetic glands.	[135]
	VIT *f* VIT *p*	Hydromethanolic ext		α-glucosidase activity.			+ Inhibition of activity in *B. forficata*. – No inhibition of activity in *B. forficata* subsp. *pruinosa*.	[78]
	VIT	Purified kaempferitrin	0.001, 0.0, 0.1, 1, 10, 100, 1000 ηM	Soleus muscle from ALX diabetic and normal rats.	M		+ Stimulatory effect of glucose uptake in muscle from normal rats. + Increase in glycogen content in muscle from diabetic rats. + Stimulation glycogen synthesis in muscle from normal rats. + Increment in protein synthesis in muscle from normal rats.	[136]
	VIT	Hydromethanolic ext		α-glucosidase activity.			± Weak inhibition of activity.	[83]
	VIT	Hexane ext Ethanolic ext		α-glycosidase, α-amylase and lipase activity. method.			+ Inhibition of enzyme activity. + Antiglycation activity.	[86]
	VIT	Ethanolic ext fra		α-glycosidase, α-amylase and lipase activity. BSA/FRU, BSA/MGO and Arg/MGO methods.			+ Inhibition of enzyme activity. + Antiglycation activity.	[89]
	HUM/O/10m	Aqueous infusion (tea)	A dessert spoon of grounded leaves in water, 3 doses	Type 2 diabetes volunteers (n = 26) and diabetic control group (n = 29).	F-M	148.70 mg/dL 154.35 mg/dL	– No hypoglycemic activity. – No reduction in glycated hemoglobin values. – No changes in body mass index values. + No changes in serum creatinine and cortisol concentration.	[137]
	HUM/ O/3m *p*	Aqueous infusion, containing rutin and trigonelline	0.15% *w*/*v* (containing rutin 2.80 μg/mL and trigonelline 2.87 μg/mL), 3 doses	Type 2 diabetes volunteers (n = 11) and prediabetic volunteers (n = 4).	F-M	X = 155.57 md/dL	+ Reduction in glycated hemoglobin values. – No hypoglycemic activity. – Increase in diuresis. – No correlation between weight and glycemia.	[138]
	HUM/O/3m	Aqueous infusion, containing rutin and trigonelline (tea)	0.4% *w*/*v* (containing rutin 1.02 mg and trigonelline 4.30 mg, in 200 mL), 2 doses	Type 2 diabetes mellitus volunteers (n = 25).	nd	X = 268 md/dL (post-prandial)	+ Reduction in glycated hemoglobin values. – No reduction in postprandial glycemia. + Reduction in triglycerides and total cholesterol levels (not clinically significant). – No changes in body weight.	[139]
*hol*	VIV/O/21d	Aqueous ext	400 mg/kg	STZ diabetic rats and normal rats.	F	>300 mg/dL	– No hypoglycemic activity in normal and diabetic rats. – No improvement in glucose tolerance in normal and diabetic rats. + Reduction in HDL-cholesterol levels in diabetic mice. – No reduction triglycerides, cholesterol and VLDL levels. – No reduction in water and food intake. + Reduction in total protein levels. – Hepatic toxicity: reduction in body weight and increment of aspartate and alanine aminotransferase activities.	[140]
	VIV/O/14d VIT/ VIV	Ethanolic ext	400 mg/kg	STZ diabetic mice and normal mice.	nd	>250 mg/dL	+ Hypoglycemic activity in diabetic mice. + Improvement in glucose tolerance in diabetic mice. + Increment of hepatic glycogen. – No changes in muscle glycogen. + Activation of gene and protein expression of enzymes involved in liver and muscle glycogenesis and glucose uptake in the muscle. + Inhibition of gene and protein expression of liver gluconeogenesis enzymes. + Inhibition of α-glucosidases (α-amilase and maltase) activity in vitro and in vivo.	[141]

* Nomenclature according to published papers: *f*—*B. forficata* subsp. *forficata*; *p—B. forficata* subsp. *pruinosa*; *c—B. candicans*. A—acute; Arg—arginine; AXL—alloxan-induced; BSA—bovine serum albumin; d—day; ext—extract; fra—fraction; FRU—frutose; HUM—clinical study in humans; IV—intravenous administration; MGO—methylglyoxal; m—month; nd—no data; O—oral administration; STZ—streptozotocin-induced; VIT—in vitro study; VIV—in vivo study.

**Table 4:** Chemical constituents from austral South American *Bauhinia*. Taxa and compound nomenclature according to published papers. *che*—*cheilantha*; *for*—*forficata*; *hol*—*holophylla*; *mic*—*microstachya*; *ruf*—*rufa*; *ung*—*ungulata*; *uru*—*uruguayensis*.

Spp	Chemical Constituents	References
*che*	**Flavonoids**: Present but not characterized **Other compounds**: α-copaene, α-guaiene, α-gurjunene, α-humulene, α-muurolol, α-pinene, α-terpineol, α-ylangene, β-colacorene, β-elemene, β-gurjunene, β-pinene, δ-cadinene, δ-elemene, λ-cadinene, λ-eudesmol, λ-muurolene, 1-epi-cubenol, 2,3-dihydro-farnesol, allo-aromadendrene, aromadendrene, bicyclogermacrene, bulnesol, camphene, caryophyllene, cubenol, (E)-bisabol-11-ol, (E)-caryophyllene, elemol, germacrene D, globulol, humulene epoxide II, limonene, maaliol, myrcene, phytol, sabinene, spathulenol, terpinen-4-ol, trans-b-guaiene, trans-isolongifolanone, tricyclene, viridiflorene, viridiflorol	[61,142]
*for*	**Flavonoids**: aromadendrin, catechin, epicatechin, eriodictyol, gallocatechin, hispidulin, isoquercitrin, isorhamnetin-3-O-glucoside, isorhamnetin-3-O-rhamnosyl rutinoside, isorhamnetin-3-O-rutinoside, kaempferol, kaempferol-3-(2/3/4-di-rhamnosyl) glucoside, kaempferol-3-O-(2-rhamnosyl) glucoside-7-O-rhamnoside, kaempferol-3-O-(2-rhamnosyl) rutinoside, kaempferol-3-O-(2-rhamnosyl) rutinoside-7-O-rhamnoside, kaempferol 3-O-(4-O-p-coumaroyl) glucoside, kaempferol-3-O-(α)-glucoside-(1′′′6′′)-rhamnoside-7-O-(α)-rhamnoside, kaempferol-3-O-[α-L-rhamnopyranosyl-(1→6)-β-D-glucopyranosyl]-7-O-α-L-rhamnopyranoside, kaempferol-3-O-dirhamnoside, kaempferol-3-O-glucoside, kaempferol-3-O-rhamnoside, kaempferol-3-O-rhamnosyl rutinoside, kaempferol-3-O-robinoside, kaempferol-3-O-rutinoside, kaempferol-3-O-rutinoside-7-O-rhamnoside, kaempferol-3-rhamnoside, kaempferol-7-O-glucoside, kaempferol-7-O-(α)-rhamnoside, kaempferol-7-O-α-L-rhamnopyranoside, kaempferol-37-O-(α)-dirhamnoside (kaempferitrin), kaempferol-arabinoside- rhamnoside, liquiritigenin, luteolin-C-hexoside, myricetin, myricetin-3-O-arabinopiranoside, myricetin-3-O-galactoside, myricetin-3-O-rhamnoside, myricetin-O-(O-galloyl)-hexoside, myricetin-O-(O-galloyl)-hexoside epigallocatech-(48) epicatechin, naringenin, naringin, quercetin, quercetin-O-arabinoside, quercetin-3-arabinoside, quercetin-3-O-(2-rhamnosyl)rutinoside-7-O-rhamnoside, quercetin-3-O-α-L-pyranoside, quercetin-3-O-[α-L-rhamnopyranosyl-(1→6)-β-D-glucopyranosyl]-7-O-α-L-rhamnopyranoside, quercetin-3-O-hexoside (isoquercetin), quercetin-3-O-galactoside, quercetin-3-O-rhamnoside (quercitrin), quercetin-3-O-rhamnosyl rutinoside, quercetin-3-O-rutinoside-7-O-rhamnoside, quercetin-3-O-rutinoside (rutin), quercetin 3-rutinoside-7-rhamnoside, quercetin-37-di-O-α-L-rhamnopyranoside, quercetin-37-di-O-rhamnoside, quercetin-O-hexoside, quercetin-O-(O-galloyl)-hexoside, quercitrin, taxifolin-3-O-rhamnoside **Other compounds**: 2,4,6-trihydroxy-octadecadienoic acid, α-bisabolol, α-bulnesene, α-cadinol, α-copaene, α-humulene, α-pinene, β-caryophyllene, β-elemene, β-ocimene, β-pinene, ß-sitosterol, γ-elemene, λ-elemene, benzyltartaric acid, bicyclogermacrene, caffeic acid, caryophyllene oxide, chlorogenic acid, copaene isomer, dihydroxyhexadecanoic acid, eicosane, epi-α-muurolol, ellagic acid, epigallocatechin-(4,8)epicatechin, eriodictyol, ferulic acid, gallic acid, germacrene, globulol, hispidulin p-coumaric acid, hydroxy-octadecatrienoic acid, isophytol, protocatechuic acid, rosmarinic acid, sabinene, salicylic acid, sinapic acid, spathulenol, syringic acid, trans-caffeic acid, trihydroxyphenanthren-2-glycoside, umbelliferone, vanillic acid, (Z)-β-farnesene, (Z,E)-farnesol, (Z,Z)-farnesol	[35,69,78,80,82,83,85,88,89,125,131,143,144,145,146,147]
	*subsp. pruinosa* **Flavonoids**: isorhamnetin-3-O-rutinoside, kaempferol, kaempferol-3-(2/3/4-di-rhamnosyl) glucoside, kaempferol-3-O-(2-rhamnosyl) rutinoside, kaempferol-3-O-robinoside, kaempferol-3-O-rutinoside, kaempferol-37-dirhamnoside (kaempferitrin), myricetin-3-O-arabinopiranoside, quercetin, quercetin-3-O-rutinoside (rutin), quercetin-3-O-(2-rhamnosyl) rutinoside, quercetin-3-O-(2/3/4-di-rhamnosyl) glucoside, quercetin-37-di-O-rhamnoside **Other compounds**: trigonelline	[78,81,84,138,139,148,149,150]
	*B. candicans* **Flavonoids**: kaempferol-3-O-β-D-glucopyranosyl-(6→1)-β-L-rhamnopyranosyl-7-O-α-L-rhamnopryranoside, kaempferol-37-O-α-L-dirhamnoside (kaempferitrin), quercetin-37-O-α-L-dirhamnoside, quercetin-3-O-β-D-glucopyranosyl-(6→1)-β-L-rhamnopyranosyl-7-O-α-L-rhamnopyranoside	[68,135]
*hol*	**Flavonoids**: 3-O-substituted flavonol, isorhamnetin, kaempferol-O-pentoside, luteolin, luteolin-deoxyhexose, myricetin-O-deoxyhexoside, myricetin-O-hexoside, myricetin-O-pentoside, quercetin, quercetin-3-O-deoxyhexoside, quercetin-3-O-hexoside, quercetin-O-deoxyhexoside, quercetin-O-hexoside, quercetin-O-pentoside, quercetin-O-xilopyranoside	[97,98,100,141]
*mic*	**Flavonoids**: catechin, kaempferol-3-O-rhamnoside, myricitrin, quercetin-3-rhamnoside (quercitrin), vitexin (apigenin 8-C-glucoside) **Other compounds**: gallic acid, hexatriacontane, methyl gallate	[15,39,151]
	**var.***massambabensis* **Flavonoids**: astragalin-2″6″-O-digallate, kaempferol-3-O-rhamnoside	[105]
*ruf*	**Other compounds**: α-amorphene, α-cadinol, α-fenchene, α-gurjenene, α-pinene, γ-cadinene, δ-cadinene, allo-aromadendrene, aromadendrene, bicyclogermacrene, cis-a-bisabolene, germacrene, globulol, lepidozenol, spathulenol, sinularene, viridiflorol	[145]
*ung*	**Flavonoids**: Fisetinidol, liquiritigenin, naringenin, quercetin, quercetin arabinofuranoside, quercitrin **Other compounds**: 2′-hydroxy-3,5-dimethoxy-4-methylbibenzyl, 2′-hydroxy-3,5-dimethoxybibenzyl, 3-O-methyl-D-pinitol, 6,9-guaiadiene, 8-α-11-elemenedio, α-cadinol, α-calacorene, α-copaene, α-cubebene, α-guaiene, α-humulene, β-bourbonene, β-caryophyllene, β-copaene, β-elemene, β-selinene, γ-cadinene, γ-ermacrene, γ-muurolene, allo-aromadendrene, betulinic acid, caryophyllene, caryophyllene oxide, cubenol, cyclosativene, (E)-caryophyllene, eleagnine, eriodictyol, glutinol, guibourtinidol, harmane, humulene epoxide, humulene epoxide II, junenol, pacharin, sitosterol, spathulenol, stigmasterol, taraxerol, taraxerone	[115,117,118,119,152,153]
*uru*	**Flavonoids**: kaempferol-3-rhamnoside, kaempferol-galloyl-rhamnoside, quercetin-3-rhamnoside (quercitrin), quercetin-galloyl-rhamnoside	[150]

## Data Availability

Not applicable.
